# Cytokine Profiles, CTL Response and T Cell Frequencies in the Peripheral Blood of Acute Patients and Individuals Recovered from Hepatitis E Infection

**DOI:** 10.1371/journal.pone.0031822

**Published:** 2012-02-22

**Authors:** Anuradha S. Tripathy, Rumki Das, Sanjay B. Rathod, Vidya A. Arankalle

**Affiliations:** Hepatitis Group, National Institute of Virology, Pune, India; Duke University School of Medicine, United States of America

## Abstract

**Background:**

Hepatitis E is a major public health problem in the developing countries. Pathogenesis of hepatitis E virus (HEV) infection is poorly understood.

**Methods:**

This case-control study included 124 Hepatitis E patients (46 acute and 78 recovered), 9 with prior exposure to HEV and 71 anti-HEV negative healthy controls. HEV induced CTL response by Elispot, cytokines/chemokines quantitation by Milliplex assay and peripheral CD4+ & CD8+ T cell frequencies by flow cytometry were assessed.

**Results:**

Among the patient categories, HEV specific IFN-γ responses as recorded by Elispot were comparable. Comparisons of cytokines/chemokines revealed significantly high levels of IL-1α and sIL-2Rα during acute phase. Circulating peripheral CD4/CD8+ T-cell subsets in acute and recovered individuals were comparable compared to controls, while among patient categories CD8+T cell subset was significantly higher in recovered individuals.

**Conclusions:**

Our findings suggest that IL-1α and sIL-2Rα play a role in the pathogenesis of acute Hepatitis E infection. Lack of robust HEV ORF2-specific CTL response in the peripheral blood of HEV infected patients during the acute and recovered phases of the disease may be associated with involvement of innate immune cells/localization of the immune events at the site of infection.

## Introduction

Hepatitis E virus (HEV) is a major cause of epidemic and sporadic acute hepatitis that is usually mild and self-resolving with the highest disease attack rate among young adults. However, a few patients develop severe disease manifesting as fulminant hepatic failure (FHF) [1]. Though the overall case-fatality rate of acute hepatitis E is 0.5–1%, it may reach up to 20–25% among pregnant women [2].The disease severity in pregnant women might be attributed to altered immune responses in these patients [3].

HEV specific IgM and IgG antibodies appear to have neutralizing activity [4], their longevity and exact role in protection against HEV re-infection remains unclear [5].Significantly higher anti-HEV IgM and IgG titers in the FHF patients bled within 2 weeks of onset of jaundice irrespective of the outcome of the disease, suggested the involvement of humoral immune response in HEV pathogenesis [6].

Peripheral blood mononuclear cells (PBMCs) serve as a reservoir for other hepatotropic viruses that cause chronic infection, such as hepatitis B virus (HBV) and hepatitis C virus (HCV) [7], [8], [9]. Detection of HEV RNA in the absence of replication in PBMCs of acute hepatitis E patients indicated that these cells are not the site for replication of HEV [10]. Chandra et al reported HEV RNA positivity in the sera after normalization of transaminases, suggesting that liver injury is independent of viral replication [11]. Recent studies have indicated the involvement of expression profiles of peripheral immune cells in the pathogenesis of acute hepatitis E [12], [13].

Previous studies have shown correlation between IFN- γ release by Elispot and severity in resolving acute hepatitis E patients [14]. However, comparative evaluation of the cytokine/chemokine profiles of the immune cells and the role of antigen-specific CTLs during HEV infection/recovery still remains to be explored.

The present study attempts to understand the involvement of host factors: HEV specific cytokine/chemokine profiles, T cell response in terms of IFN-γ release directed against ORF2 protein of HEV by Elispot assay and peripheral CD4+ & CD8+ T cells frequencies, during the acute and the recovery phases of self-limiting acute hepatitis E.

## Methods

### Study population

The study population of 204 included (1) Acute viral hepatitis (AVH-E) group: 46 patients (2) Recovered group: 78 patients (3) Exposed group: 9 individuals and (4) Control group: 71 healthy individuals, termed as HEV naive (Table 1).The diagnosis of hepatitis E was based on the presence of IgM antibodies to hepatitis E virus (IgM-anti-HEV) as detected by ELISA [15]. The patients were classified as acute viral hepatitis (AVH-E) based on the standard clinical and biochemical criteria [6]. Briefly, patients presenting with icterus, dark-colored urine, elevated alanine aminotransferase (ALT) (normal level, 4–40 IU/l) and/or bilirubin levels (>1 mg/ml) in the serum and/or presence of bile salts and pigments in the urine were considered to have hepatitis. The recovered individuals had a previous history of acute hepatitis E. The patients were enrolled from different hepatitis outbreaks in the state of Maharashtra, Western India. The exposed group consisted of healthy persons who could not recall a history of an illness resembling viral hepatitis indicating that they had been exposed to HEV in the past. They were positive for IgG anti-HEV antibodies. The patient and the exposed group population negative for HBsAg, anti-HIV, IgM anti-HAV, anti-HCV antibodies were only included in the study. The Control group/HEV naive consisted of age- and sex-matched apparently healthy individuals negative for HBsAg, anti-HIV, anti-HCV, IgM/IgG anti-HEV and IgM anti-HAV antibodies. None of the patients was having any past history of chronic liver disease, severe systemic illness or was undergoing therapy at the time of sampling. Due to logistic problems the quantity of blood sample drawn in most of the patients was small. Hence, the subjects assessed for cytokine assay, T cell frequencies and Elispot were not necessarily the same. The samples from the exposed group were processed only for Elispot assay. This study was approved by the Institutional “Ethical Committee for Research on Humans” and written consent was obtained from all the participants involved in the study.

**Table 1:** Clinical characteristics of patients and controls enrolled in the study.

Parameters	AVH-E	Recovered	Exposed	Controls/HEV naive
Study population	n = 46	N = 78	n = 9	n = 71
Age (years) median range	36(18–53)	38(21–53)	30(21–47)	25(21–45)
Sex ratio M∶F	30∶17	61∶21	6∶3	57∶21
ALT (IU/L) median (range)	179 (50–392)	20.4 (5–32)	ND	20(9–35)
IgM titre median(range)	12800(200–51200)	1600(200–6400)	ND	ND
IgG titre. median(range)	51200(1600–409600)	102400(800–409600)	150(100–200)	ND
Post Onset days of illness	12±4.5	53±5	ND	ND

### Serological & Molecular assays

Blood samples were collected in K3 EDTA tube and plasma was separated. All samples were screened in ELISA for the presence of IgM antibodies against hepatitis A virus (anti-HAV IgM; Hepavase A-96, General Biologicals Corp., Taiwan), hepatitis B surface antigen (HBsAg; Surase B-96, General Biologicals), IgM antibodies to hepatitis B core antigen (anti-HBc IgM; Anticorase B-96, General Biologicals), antibodies to hepatitis C (anti-HCV; Ortho HCV 3.0, Ortho Clinical Diagnostics, USA), antibodies to HIV-1 (INSTI™ HIV-1 antibody Test Kit, Biological Laboratories Inc, Richmond, British Columbia, Canada), anti-HEV IgM and IgG antibodies by ELISA based on the use of recombinant ORF2 antigen (rORF2p) [15] and serum alanine amino transferase levels (ALT; Span Diagnostics, India). The end points of serum ALT levels, IgM and IgG levels were determined as previously described [16].

### Preparation of ORF2 protein

Complete ORF2 gene (1983 bp:5147–7129 nt, corresponding to 660 aa) from genotype 1 of HEV was cloned in pVAX1; 56 kDa. This ORF2 protein (rORF2p) was expressed in baculovirus system (56 kDa) [17], [18].It was purified by HPLC and was used in all the assays [6].

### Preparation of peripheral blood mononuclear cells (PBMCs)

Peripheral blood mononuclear cells (PBMCs) were isolated from fresh blood collected in K3 EDTA tube by Ficoll-Hypaque density gradient centrifugation. Cells were re-suspended in RPMI 1640 medium (Invitrogen, Carlsbad, USA) supplemented with 2 mmol/l L-Glutamine, 1 mmol/l sodium pyruvate and 20 ug/ml of gentamycin.

### Enzyme-linked Immunosorbent spot (Elispot) assay

Hepatitis E virus specific Elispot assay for IFN-γ release was done in 27 acute HEV patients, 22 recovered from Hepatitis E, 9 exposed individuals to Hepatitis E(IgG anti HEV +ve) and 18 Control/HEV naïve individuals (IgG anti HEV −ve) as previously described [19].Briefly, 96-well nitrocellulose bottomed plates (MAIPS 4510; Millipore Bedford, MA) were coated with recombinant anti-human IFN-γ mAb (Mab Tech, Sweden) at a concentration of 5 µg/ml and kept at 4°C overnight. The plates were washed with wash buffer (phosphate buffered saline, (PBS) containing 0.5% (v/v) Tween 20) and blocked with fetal bovine serum (FBS) for 2–4 h at 37°C. To estimate the number of HEV-specific IFN-γ secreting spot forming cells (SFCs), 1×10^5^ PBMCs/well were stimulated with rORF2p (20 µg/ml). In the control wells, cells were stimulated with 10 ug/ml of Phytohemagglutinin A (PHA-A) (Sigma, USA)/medium alone. After incubation at 37°C, the plates were washed with PBS and with wash buffer. Biotinylated anti-human IFN-γ mAb (Mab Tech, Sweden) at a concentration of 2 µg/ml was added. After washing, avidin-bound horseradish peroxidase (Vectastain; Vector Laboratories, USA), containing 0.1% Tween 20, was added and kept at room temperature. This was followed by the addition of the AEC substrate (Sigma, St. Louis, MO). After drying, SFCs were counted in ELISPOT reader (Carl zeiss, Germany) using KS ELISPOT software and was expressed per 10^5^ cells. The cutoff level of SFCs was calculated as the average number of SFCs in the negative control wells. Assays with high background or with no PHA response were excluded. Normalization of the data was done by subtracting the number of spot forming cells in unstimulated wells from the antigen stimulated wells. The values are expressed as median (range).The patient categories were compared with the HEV naïve group. Statistical analyses were carried out in the data after normalization.

### Stimulation of PBMCs and cytokine assay

Cytokine assay was carried out in 24 well plates in 44 AVH-E patients, 23 recovered individuals and 16 controls/HEV naïve as previously described [6]. Cytokine levels were measured from culture supernatants for each sample using a Milliplex cytokine assay (Millipore Billerica, MA).

### Milliplex Analysis

Cytokine concentrations in the rORF2p stimulated culture supernatants were determined using Milliplex immunoassay Kit (Millipore Billerica, MA). The assay was performed according to the manufacturer's instructions as reported [18]. Eight cytokines (IL1α, IL1RA, sIL-2Rα, IL-12p70, TNF-β, IFN-α, MIP-1, IL-8) were estimated.

### CD4+ T and CD8+ T cell enumeration by flow cytometry

Freshly drawn 100 ul peripheral blood was stained with anti- human CD4+ T (FITC) and CD8+ T (FITC) in 46 AVH-E, 78 recovered individuals and 71 controls/HEV naïve along with appropriate isotype controls (ebiosciences, San Diego, CA, USA) and were analyzed as previously described [19]. For each experiment 10,000 events were acquired within the lymphocyte gate and were analyzed using the Cell Quest software (Becton Dickinson, USA). Results are expressed as median (range).

### Statistical analysis

The levels of cytokines and chemokines were analysed after log transformation and a value of 0.1 pg/ml was used in case of undetectable concentration in the tested samples. Mann–Whitney U-test was used for numerical data. Correlation between quantitative parameters was analyzed using Spearman rank correlation coefficient test. For all analyses, a p-value ≤0.05 derived from a two-tailed test was considered to be significant. All statistical analysis was performed with SPSS11.0 software (SPSS Inc., Chicago, IL, USA).

## Results

### rORF2p specific CTL activation in Elispot

**Figure 1:**
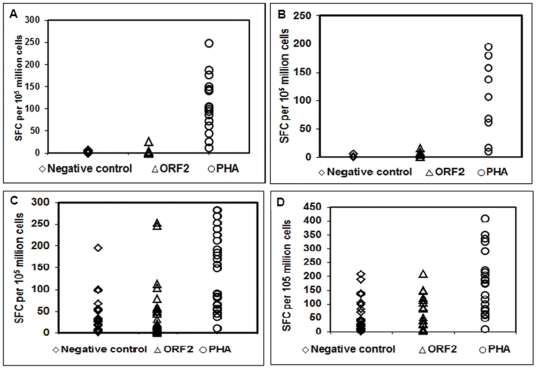
IFN-γ responses by Elispot in Hepatitis E patients and controls. Scatter plot showing Hepatitis E antigen specific IFN-γ responses in different groups. A) IgG anti HEV negative/HEV naive, n = 18 B) IgG anti HEV positive/HEV exposed, n = 9, C) AVH-E, n = 27 and D) Recovered individuals from hepatitis E, n = 22. PBMCs were isolated from all subjects mentioned and were cultured with recombinant ORF2 protein in-vitro. IFN-γ secreting cell frequencies were determined by Elispot assay. The figures in parentheses show the number of SFCs per10^5^ cells.

To characterize the antigen-specific T-cell response to HEV, we determined the frequency of IFN-γ-producing T cells in response to rORF2p by ELISPOT assay (Figure 1). In control/HEV naïve group (IgM/IgG anti HEV negative) (n = 18), IFN-γ responses in unstimulated, rORF2p and PHA stimulated cells were 0(0–4), 1(0–25) and 10-4(12–248) SFC/10^5^ cells respectively (Figure 1A). The corresponding figures in the exposed group (IgM anti-HEV negative and IgG anti HEV positive) (n = 9) were 0(0–6), 4(0–16) and 107(10–196) respectively (Figure 1B).

**Figure 2:**
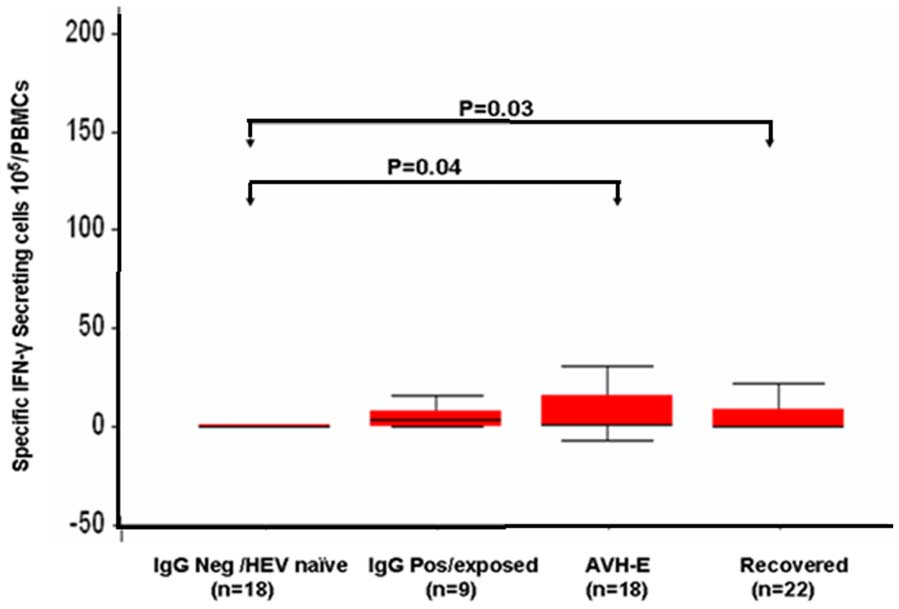
Recombinant ORF2 protein specific IFN-γ responses in all the four groups. IFN-γ Elispot response to recombinant HEV ORF2 protein in PBMC.Boxes represent interquartile ranges, vertical lines represent ranges and horizontal lines represent medians. The figures in parentheses show the number of SFCs per10^5^ cells.

In AVH-E group (n = 27), IFN-γ responses in unstimulated, rORF2p and PHA stimulated cells were 28(1–195), 22(0.6–253) and 92(11–284) SFC/10^5^ cells respectively (Figure 1C).In the recovered group (n = 22), the corresponding figures were 32(0–208), 41(2–209) and 175(10–410) respectively (Figure 1D). After normalization of the data, IFN-γ responses in the rORF2p stimulated cells of AVH-E patients and recovered individuals were significantly higher compared to controls. Among the patient categories HEV specific IFN-γ responses were comparable. The results are expressed as median (range) (Figure 2).

CTL response by Elispot was analyzed longitudinally in 6 patients at two time points, during the acute and recovery phase. One of the patients had a fall; a rise was observed in another, while in the rest 4, the SFC/10^5^ cells were in the same range (data not shown).

### Peripheral T lymphocyte population in patients and controls

The proportions of circulating peripheral CD4+ and CD8+ T cells are expressed as percentage of gated lymphocytes. In the 71 controls/HEV naïve, the median (range) of CD4+ & CD8+ T cells were 36(10.3–59.5) and 23.3(11.2–80.8) respectively. Similarly, in 46 AVH-E & 78 recovered individuals, CD4+ T cells were 35.2(10.5–79.9) & 37.3(20.4–72.8) and CD8+ T cells were 24(11.7–42.5) and 27.5(13.4–52.9) respectively. CD4+ and CD8+ T subsets were unchanged in AVH-E and recovered individuals compared to the controls. Among the patient categories, CD8+ T subset was significantly higher in recovered individuals (p = 0.04).

### Cytokine profiles during Hepatitis E infection and in recovery

**Figure 3:**
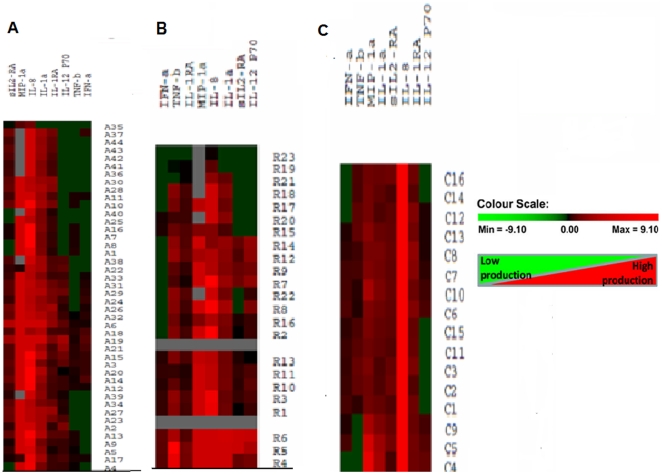
Chemokine/cytokine levels in HEV patients and controls. Cytokine/chemokine expression profiles of hepatitis E specific PBMCs in AVH-E patients, recovered individuals and healthy controls using Milliplex immune panel by 2-way hierarchical clustering. Each colored cell in the 3 hit maps represents the relative levels of expression of a particular cytokine/chemokine in a subject. Green indicates low production and red indicates high production of cytokines/chemokines. Values for eight cytokines were hierarchically clustered on log transformation. The corresponding cytokine of each cluster is listed by a cytokine symbol on the top side of the images. A) shows AVH-E patients denoted as A1–A44, B) shows recovered individual denoted as R1–R23 and. C) Shows cytokine expression of Controls denoted as C1–C16.

The levels of IL-1α, MIP-1α in AVH-E and MIP-1α in the recovered group were significantly elevated compared to controls (p<0.05 in each). Levels of IFN-α and TNF-β were significantly lower in the AVH-E group and that of sIL-2Rα, IL-1RA and TNF-β were lower in the recovered group in comparison to controls (P<0.05 in each). The levels of IL-12p70 & IL-8 were comparable in both the AVH-E & recovered groups, whereas sIL-2Rα, IL-1RA in AVH-E and IL-1α levels in recovered group were comparable with control. Comparison among the patient categories revealed significantly higher levels of sIL-2Rα and IL-1α in AVH-E (p = 0.0001 & 0.018 respectively) (Figure 3A, 3B & 3C).

### Correlation of anti HEV IgM & IgG levels with CTL response

Spearman analysis showed that for AVH-E patients, IFN-γ release by rORF2p in Elispot was negatively correlated with IgG levels (r = −0.738, p = 0.015). No correlation of IgM anti-HEV titers with IFN-γ release by Elispot was recorded.

## Discussion

The present study is a comprehensive report demonstrating the nature of involvement of (a) recombinant ORF2 protein specific CTL response (b) cytokine profiles directed against recombinant ORF2 protein and (c) peripheral T lymphocytes in the pathogenesis/recovery from acute hepatitis E.

The outcome of HEV infection is reported to be dependent on both the T cell subsets and antigen non-specific inflammatory cells recruited to the liver [12]. In our series, the percentage of CD4+ & CD8+ T cells were unchanged in AVH-E and recovered individuals compared to the controls. CD8+ T population was significantly more in the recovered individuals compared to AVH-E. Prabhu et al. showed the presence of more number of CD4+ T cells in the liver of FHF caused by HEV when compared to that of healthy individuals elucidating the role of CD4+ T cells in providing help and activating cytotoxic T cells [20]. Trehanpati et al. reported an increase in the proportion of CD8+ T cells in AVH-E but no change in the CD4+ T cell compartment in peripheral blood [13]. The difference in the results of these studies may be related to large differences in sample size and the difference in cluster of differentiation (CD) markers used.

The involvement of CD8+ T cells in the pathogenesis and virus clearance in acute infections with hepatitis A, hepatitis B and hepatitis C virus has been well recognized [21], [22], [23]. Hence, CTL responses directed against HEV protein was assessed to understand its role in the host cell injury. Significantly high HEV specific and robust nonspecific IFN-γ producing T-cell response in Elispot in AVH-E patients of current series suggests role of IFN-γ in the clearance of HEV infection. Diminished HEV specific and robust nonspecific IFN-γ-producing T-cell response in recovered cases compared to controls makes its role unclear Further work is needed to determine if this response involves CD4+ or CD8+ subsets (or both). IFN-γ is the signature cytokine that enhances the activity of NK cells. High levels of background IFN-γ production in the absence of HEV specific protein in both the patient categories goes in parallel with the previous report of involvement of innate mechanisms involving NK/NKT/T reg cells in the pathogenesis of hepatitis E [24].

Wu et al. reported that acute hepatitis patients displayed a detectable HEV specific T cell response, whose levels decreased along with the decreasing titre of anti-HEV antibody and in convalescence [14]. A study by Srivastava et al. reported a heightened IgG response as assayed by B cell Elispot to be associated with a more severe disease [25]. In the current series of acute and recovered individuals, IgM levels did not have any correlation with HEV specific CTL response; whereas IgG levels of the acute patients were negatively correlated with the same. In spite of the contradictory reports due to difference in assays, post onset days of illness of study subjects, the involvement of IgG levels in HEV pathogenesis cannot be overlooked. Similar findings were reported by Husain et al. reporting increased frequency of recombinant ORF2 protein specific IFN- γ secreting cells in acute hepatitis E patients compared to the controls elucidating reactivity of T cells to the full length ORF2 protein [26].

Elevated serum levels of IL-1α and IL-1β have been correlated with the impairment of hepatocytes, since they were reported to be present only in the acute and not in the recovery phase of viral hepatitis [27]. Similarly, increased IL-1α levels in AVH-E vs recovery in the current series of patients and increased levels of IL-1β and TNF-α in AVH-E and recovered cases in our previous report [16] suggest their probable role in the pathogenesis of hepatitis and hepatocytic injury. In acute hepatitis patients, high levels of sIL-2Rα returning to normal during resolution has been suggested to be associated with the diagnosis of inflammation in hepatitis, a process in which interleukin 2 may participate [28]. With our data of higher levels of sIL-2Rα in AVH-E patients vs. recovery, its use as a marker of inflammation in hepatitis could further be ascertained with supporting data from patients with fulminant hepatitis E.

In conclusion, our data suggest that there is no robust HEV ORF2-specific T cell response in the peripheral blood of HEV infected patients during the acute and recovered phases of the disease. Further work assessing HEV specific CTL responses in the peripheral blood of acute, recovered individuals and at the site of infection of fulminant hepatic failure patients by longitudinal study at different post onset days of illness remains to be elucidated.
